# Public Health Response to Imported Case of Poliomyelitis, Australia, 2007

**DOI:** 10.3201/eid1511.090027

**Published:** 2009-11

**Authors:** John A. Carnie, Rosemary Lester, Rodney Moran, Lynne Brown, Julian Meagher, Jason A. Roberts, Bruce R. Thorley

**Affiliations:** Department of Human Services, Melbourne, Victoria, Australia (J.A. Carnie, R. Lester, R. Moran, L. Brown, J. Meagher); Victorian Infectious Diseases Reference Laboratory, Melbourne (J.A. Roberts, B.R. Thorley)

**Keywords:** Poliomyelitis, wild poliovirus type 1, polio vaccine, inactivated polio vaccine, importation, viruses, synopsis

## Abstract

Australia, along with 36 other countries in the Western Pacific Region, was declared free of poliomyelitis by the World Health Organization in October 2000. Yet, the persistence of wild poliovirus in the 4 remaining polio-endemic countries—Afghanistan, India, Nigeria, and Pakistan—poses a risk for its importation into all countries declared polio free. We describe the public health response and outcomes resulting from the importation of a wild poliovirus infection in Melbourne, Australia, in July 2007. This response, based on an assessment of the risk for transmission, included offering vaccination with inactivated polio vaccine to the contacts and placing the index patient in isolation and the household contacts in quarantine until consecutive fecal specimens were negative for poliovirus by culture. The experience gained from the polio importation event in Australia may assist other polio-free countries to prepare for, and respond to, a similar event. No secondary clinical cases resulted from this importation.

## Abstract 2

Inactivated polio vaccine was offered, and the index case-patient and household contacts were quarantined.

Since 2006, wild poliovirus has been endemic in only 4 countries: Afghanistan, India, Nigeria, and Pakistan. Although many countries have not reported a case of poliomyelitis caused by wild poliovirus for some years, they remain at risk for importation of the disease. Australia and the other countries of the Western Pacific Region were declared polio free in October 2000 (*1*). However, the last reported case of wild poliovirus infection in Australia was imported from Turkey in 1977 (*2*). National departments of health in this region must remain vigilant for such an event and respond appropriately to reduce the risk for local transmission.

The World Health Organization (WHO) Global Polio Eradication Initiative recommends clinical surveillance for cases of acute flaccid paralysis in children <15 years of age and suspected paralytic polio in a person of any age as the most sensitive means of detecting a case of imported poliomyelitis in countries declared polio free (*3*). The Australian government initiated surveillance for acute flaccid paralysis, following the WHO guidelines, in 1995. WHO established a global polio laboratory network, which includes a Polio Regional Reference Laboratory in Australia, to confirm poliovirus infection because other conditions manifesting acute paralysis can mimic polio. Nevertheless, Australia’s ability to detect and respond effectively to the importation of a wild poliovirus infection has been questioned because gaps have occurred in surveillance for acute flaccid paralysis surveillance and in the referral of fecal specimens for laboratory testing (*4*).

Australia began exclusive use of inactivated polio vaccine (IPV) in place of the Sabin oral polio vaccine on November 1, 2005. In Victoria in 2007, the proportion of children who received at least 3 doses of polio vaccine was 92.8% at 12 months of age and 95.9% at 2 years of age. Coverage with at least 4 doses of polio vaccine was 91.4% at 6 years of age. No reliable data exist on vaccination coverage with polio vaccine in the adult population.

We describe the public health response to an importation of wild poliovirus infection that occurred in Melbourne, Australia, in July 2007; the last reported case of polio in Australia was in 1977 (*2*). The issues addressed as a result of this event would be similar for other countries, and the lessons learned may be incorporated into national planning for a polio outbreak (which requires only a single confirmed case).

## Notification of the Index Case

On July 7, 2007, the Department of Human Services (DHS) in Victoria, Australia, was notified of a suspected case of imported poliomyelitis in a 22-year-old man (a university student). The patient, who was studying in Melbourne, had returned home to Pakistan on March 13, 2007, and in early June, he visited Islamabad and the North-West Frontier Province. On June 22, 2007, fever, nausea, and pain in the lower back and legs developed and progressed to lower limb weakness. The symptoms appeared to resolve, and he returned to Melbourne, arriving on a flight from Bangkok, Thailand, on July 2, 2007. However, he remained at home with back pain and lower limb weakness and consulted a general practitioner on July 6, 2007. He was advised to go to a hospital, where the emergency department admitted him for further investigation. The patient reported receiving at least 3 doses of oral poliomyelitis vaccine as a child.

A case report describing the clinical features and treatment of the patient and the initial laboratory investigation was published by Stewardson et al. (*5*). Briefly, a magnetic resonance image of the patient’s spine, performed on July 7, indicated increased signal in the anterior horn cells, which is highly suggestive of poliomyelitis. The patient was placed in a single room with enteric precautions, and DHS was notified of the diagnosis of poliomyelitis. Although an initial pan-enterovirus reverse transcription–PCR (RT-PCR) performed directly on a fecal specimen collected on July 7 was reported as negative, the National Polio Reference Laboratory confirmed the diagnosis of poliomyelitis by isolation of non–Sabin-like poliovirus type 1 on July 13. The virus was subsequently reported to have high nucleic acid sequence identity with wild poliovirus isolates from Pakistan, which provided an epidemiologic link with the patient’s travel history. The index patient was placed in isolation in the hospital until 2 successive fecal specimens, collected 1 week apart, were negative for poliovirus by viral culture and RT-PCR (a total of 34 days).

## Public Health Response

DHS coordinated the public health response in the state of Victoria, while national and international responsibilities were handled by the Australian government Department of Health and Ageing. At the national level, this included liaising with the Communicable Diseases Network of Australia, the Australian Health Protection Committee, and the Public Health Laboratory Network. The case was one of the first reported to WHO under the International Health Regulations (2005), which came into effect in June 2007 and require member countries to notify WHO of poliomyelitis cases (*6*). On confirmation of the diagnosis of poliomyelitis, DHS performed a risk assessment for the potential infection of contacts of the index patient with wild poliovirus. Contacts were grouped as the following: 1) close contacts who resided with or visited the index patient’s residence, 2) fellow passengers on the airplane from Bangkok to Melbourne, 3) public contacts and staff at the general practitioner’s clinic, and 4) public contacts and healthcare workers (HCWs) at the hospital (Table 1).

**Table 1:** Summary of the public health response and the outcomes to the importation of wild poliovirus, Australia, July 2007*

"I would like to expand my network with women of substance while keeping it classy and away from the usual club scene."		
Persons investigated	Response	Outcome
Index patient	Isolated in hospital after magnetic resonance image was suggestive of poliomyelitis.	Discharged when 2 fecal specimens, collected at least 7 d apart, were negative for enterovirus by cell culture and RT-PCR (total of 34 d).
Household contacts	5 housemates, 1 visitor, and the housekeeper received IPV and placed in home quarantine under a Public Health Order. Recommend serum collection before vaccinating contacts to test for IgM against polio. Another friend who visited the residence of the index case was boosted with IPV only.	Home quarantine lifted when 2 fecal specimens, collected 24–48 h apart, were negative for enterovirus by cell culture and RT-PCR. Housemates required support to ensure compliance, which included grocery deliveries, bill payments, and a range of other assistance.
Airplane contacts	Media release informing public of imported case of polio and offer of vaccination for persons who disembarked in Melbourne. DHS provided with 235 Passenger Declaration cards of persons who disembarked in Melbourne. DHS undertook contact tracing of airplane passengers (Table 2). One teenage passenger hospitalized with fever and diarrhea. 10 persons not on the airplane manifest were vaccinated as their details could not be readily determined; 7 airport workers who cleaned the plane were vaccinated with IPV.	Hospitalized passenger: single CSF and 3 fecal specimens (collected more than 24 h apart), were tested for enterovirus; CSF positive for enterovirus RNA by RT-PCR; all other tests negative by cell culture and RT-PCR.
Medical clinic contacts	14 staff members and 81 patients initially regarded as potentially at risk for exposure Nine staff identified as at risk and offered vaccination with IPV. 24 patients and 6 relatives/friends identified as at risk and offered vaccination with IPV. Letters sent to a further 8 recommending vaccination. Adult patient later hospitalized with fever, gastrointestinal illness and general weakness and spouse had respiratory illness. Upon discharge, they were asked to remain at home pending specimen results. 7-y-old child was later hospitalized with seizures.	Adult admitted to hospital and spouse: 2 fecal specimens, collected more than 24 h apart, negative for enterovirus by cell culture and RT-PCR. Child who was hospitalized: 1 CSF and a fecal specimen tested for enterovirus; CSF positive for enterovirus RNA by RT-PCR, fecal specimen negative for enterovirus by RT-PCR and cell culture.
Contacts at Box Hill Hospital	102 patients and 63 relatives/friends from either the Emergency Department or the Ward were identified as at risk: 17 were not contactable; 37 were vaccinated by their own doctors; 83 HCWs were identified as at risk and vaccinated with IPV. Identification of 9 overseas-born HCWs without evidence of recent polio vaccination.	Symptomatic HCW with back pain: single fecal specimen negative for enterovirus by RT-PCR and cell culture. HCWs without evidence of recent polio vaccination: 2 fecal specimens, collected more than 24 h apart, negative for enterovirus by RT-PCR and cell culture.

*IPV, inactivated polio vaccine; RT-PCR, reverse transcription–PCR; Ig, immunoglobulin; CSF, cerebrospinal fluid; HCW, healthcare worker.

Household contacts were judged to be at highest risk. Anyone who used the same toilet as the index patient before it had been cleaned was regarded as being at lesser risk, especially because the patient had not used a toilet to have a bowel movement either on the plane, at the general practitioner’s clinic, or at the hospital emergency department. In virtually all instances, the vaccination history of contacts was uncertain. Although the likelihood of transmission was deemed to be low, a cautious approach to the situation led to a comprehensive public health response.

### Household Contacts

Household contacts of the index patient were identified as his 5 housemates, 1 visitor who had stayed overnight after the index patient’s return from overseas, and a housekeeper who cleaned the index-patient’s premises, but did not reside there. The household contacts were placed under a public health order following the state’s health laws to remain in home quarantine until released by DHS. The 5 housemates were quarantined at their principal place of residence, together with the visitor who joined them. The housekeeper was quarantined in her own house. The contacts were provided with information on poliomyelitis and given booster doses of IPV. Doses of IPV were administered subcutaneously, according to the Australian immunization guidelines (*7*), thus avoiding the potential for provocation poliomyelitis (*8*). In hindsight, serum collection from close contacts before booster vaccination would have enabled testing for immunoglobulin M against poliovirus to assess the risk for transmission of wild poliovirus due to asymptomatic infection. All household contacts remained in quarantine until 2 fecal specimens, collected >24 hours apart, were negative by viral culture and RT-PCR (a total of 16 days) (Table 1).

### Airplane Contacts

The index patient reported that he had used the toilet on the flight from Bangkok to Melbourne, although only to urinate. Although the risk to the fellow passengers was deemed to be low, contact tracing was instituted for the passengers on the flight. Two hundred thirty-five passengers terminated their journey in Melbourne (a few passengers went on to other areas in Australia), and their incoming passenger cards were obtained by DHS through the Department of Health and Ageing. Upon laboratory confirmation of the diagnosis of the traveler’s poliomyelitis, a media bulletin was released by DHS on July 14, advising the public of the case and asking passengers from the flight to contact a national public health telephone line to obtain further information and to receive a booster polio vaccination. Media interviews were conducted by DHS staff, and a health alert was issued for hospitals in Victoria.

DHS also contacted the airplane passengers directly by telephone, letter, or email to provide information on poliomyelitis and offer a single booster dose of IPV, regardless of previous poliomyelitis vaccination history. The results of the airplane contact tracing are shown in Table 2. Seven airport workers responsible for cleaning the plane used by the index patient were also given IPV by DHS, while the Department of Health and Ageing agreed to undertake follow-up of the flight crew.

**Table 2:** Outcome of tracing the 235 airline passengers who arrived in Melbourne, Australia, on the same flight as the index patient with polio, 2007*

Action	No. passengers
Vaccinated with IPV by DHS	77
Preferred vaccination by own doctor	96
Refused vaccination	4
Recently vaccinated against polio	3
Contacted by letter	14
Contacted by email	15
Subtotal	209
Incorrect email or mail address	12
Illegible incoming passenger cards	14

*IPV, inactivated polio vaccine; DHS, Department of Health Services.

### Contacts at the Medical Clinic

On July 6, 2007, the index patient consulted a general practitioner about the symptoms that recurred after his arrival in Melbourne on July 2. He later informed DHS that he had used a toilet at the clinic to pass urine only and so the same risk assessment criteria were used as for the airplane contacts. Nine healthcare staff, 24 patients, and 6 of their friends or relatives were administered IPV.

### Hospital Contacts

We identified persons who may have used a toilet at the hospital Emergency Department and on the ward where the index patient stayed before isolation procedures were instituted, and we recommended that they receive IPV (Table 1). In total, 37 hospital patients or their friends or relatives were administered IPV by their own doctors, and 3 had recently received their routine childhood vaccinations.

HCWs who possibly had contact with the index patient were regarded as at risk, and a total of 83 hospital staff members each received 1 dose of IPV. Australian-born HCWs were judged likely to have been fully immunized and therefore less likely to be at risk. Nine overseas-born hospital staff members who could not provide evidence of vaccination or a booster dose within the last 10 years, per the Australian immunization guidelines (*7*), were requested to provide 2 fecal specimens, at least 24 hours apart, for virus culture.

### Symptomatic Contacts

Three symptomatic cases that required hospitalization were identified during contact tracing (Table 1). A teenager on the same flight from Bangkok as the index patient was hospitalized with fever and diarrhea, and a 7-year-old child who attended the same general practitioner’s clinic as the index patient was admitted with seizures. Cerebrospinal fluid (CSF) collected from both patients was positive for enterovirus RNA by RT-PCR, but fecal specimens were negative by cell culture and RT-PCR. The enteroviruses detected in the CSF from both patients were not identified. In addition, a man who attended the general practitioner’s clinic was hospitalized with fever, gastrointestinal illness, and general weakness. Two fecal specimens were collected from the patient and also from his spouse, who was having a respiratory illness, as a precautionary measure. The couple was asked to remain at home in voluntary quarantine until laboratory testing of the specimens was completed. All fecal specimens were reported as negative for enterovirus by cell culture and RT-PCR. One hospital HCW who had contact with the index patient exhibited backache, but a fecal specimen was negative for enterovirus by RT-PCR and cell culture.

### Cleaning and Disinfection

Lastly, the issue arose of cleaning and disinfection of toilets used by the index patient. Survival of poliovirus is favored by lower temperatures and high relative humidity. The virus can survive outside the human body for weeks at room temperature (*9*). Effective disinfectants include sodium hypochlorite, glutaraldehyde, or formaldehyde solutions. The WHO Guide to Hygiene and Sanitation in Aviation (*10*) indicates that the correct use of sodium hypochlorite is to apply a solution of 100 mg/L and keep it in contact with surfaces for 30 minutes; then the surfaces should be rinsed with warm water and dried.

## Discussion

The 2007 wild poliovirus importation generated widespread media coverage around Australia that assisted with contact tracing. However, tracing the passengers who disembarked with the index patient in Melbourne was difficult because of poor handwriting and inaccurate information on many of the arrival cards. This experience has implications for the urgent tracing of persons potentially exposed to other infectious diseases of public health significance, such as pandemic influenza. Despite extensive contact tracing, 26 (11%) of the 235 passengers could not be contacted. For the 96 passengers who chose to see their own doctor for vaccination and the 29 passengers who were contacted by letter or email, outcome is not known.

In large part, the laboratory investigation of the index patient and household contacts followed the procedures recommended by WHO for routine acute flaccid paralysis surveillance with collection of 2 fecal specimens obtained 24–48 hours apart, due to intermittent virus shedding, for virus culture (*11*). Virus cell culture was accepted by DHS as the approved standard for the test procedures, in agreement with the recommendation by WHO. This proved decisive because, for the first fecal specimen, enterovirus was not detected by RT-PCR performed directly on the specimen (*5*). RT-PCR is still a powerful tool for enterovirus detection as exemplified by the test results for the 2 positive CSF specimens. The testing, by cell culture and RT-PCR, of specimens from persons with suspected poliomyelitis and their contacts is recommended. This ensures that test results are determined by using the most rapid and sensitive methods available.

Household contacts of case-patients are at high risk of infection (*12*). We recommended that they be quarantined at home and that stool specimens be collected a minimum of 3 days after the first contact with the index patient to allow sufficient time for an infection to become established. As excretion of poliovirus in the feces can continue for several weeks (*13*,*14*), we sought advice from WHO and the Centers for Disease Control and Prevention (Atlanta, GA, USA) regarding when the index patient and the household contacts could be released from isolation and home quarantine, respectively. The criteria accepted by DHS was for 2 fecal specimens, collected 7 days apart for the index patient and >24 hours apart for the household contacts, to be negative for poliovirus isolation by cell culture (M. Pallansch, S. Roesel, pers. comm.).

The time taken to determine a negative result by cell culture leads to an inevitable delay in finalizing patient tests, which, in the circumstances described in this report, had implications for when the index patient and household contacts could be released from hospital isolation and home quarantine, respectively: 34 days for the index patient and 16 days for the household contacts. It should be noted that household quarantine of the contacts required substantial logistical support by DHS staff in terms of food and entertainment. This also has implications for large-scale quarantine as would be required in an influenza pandemic.

No published evidence is available on the role of polio vaccine as postexposure prophylaxis against paralytic disease. However, in persons with some preexisting immunity, which would include most of the Australian population, boosting the immune response with a single dose of oral polio vaccine or IPV is likely to reduce both pharyngeal and intestinal excretion of poliovirus in those who have been infected (*15*). The extent to which one undertakes tracing of contacts who used the same toilet was extensively debated by the Communicable Diseases Network Australia and the Australian Health Protection Committee. DHS opted to invite nonhousehold contacts to come to departmental offices or see their own physician for a booster dose of IPV and to be given information on the disease and its symptoms and signs as a precautionary measure. Booster doses of IPV have also been recommended for HCWs who have close contact with patients who might be excreting wild poliovirus (*15*). We followed this advice with the HCWs involved with the index patient, but issues arose in relation to the lack of immunization records, particularly with some of the overseas-born HCWs. We now advise that HCWs in close contact with an index patient with poliomyelitis, who have no recorded immunization history or who are not fully vaccinated, should provide 2 fecal specimens collected 24–48 hours apart and complete a course of vaccination with IPV, in accordance with the current Australian immunization guidelines (*7*).

## Conclusions

Until this imported case, poliomyelitis caused by wild poliovirus had not been reported in Australia for 30 years. The case necessitated a rapid and extensive public health response. The age and vaccination history of the index patient highlight the need for public health officials worldwide to prepare for imported cases of suspected polio in persons of any age and with prior vaccination. The experience gained from the public health response to the importation in 2007, particularly in relation to tracing of contacts, isolation of cases, and quarantine of close contacts, has been incorporated into the national outbreak response plan for the investigation of cases of acute flaccid paralysis and suspected polio, published by the Australian government’s Department of Health and Ageing (*16*).
